# Online risk exposure and anxiety among college students in China: The chain mediating role of negative attribution and interpersonal security

**DOI:** 10.1371/journal.pone.0319700

**Published:** 2025-03-19

**Authors:** Haihong Wang, Shang Zhang, Na Sun, Senqing Qi, Xinfa Yi

**Affiliations:** 1 Faculty of Education, Shaanxi Normal University, Xi’an, China; 2 Mental Health Education Center, Northwest University, Xi’an, China; 3 Key Laboratory of Modern Teaching Technology, Ministry of Education, China at Shaanxi Normal University, Xi’an, China; 4 SNNU Branch Center of National Collaborative Innovation Center of Assessment Toward Basic Education Quality, Beijing Normal University, Xi’an, China; National Institutes of Health, University of the Philippines Manila / De La Salle University, PHILIPPINES

## Abstract

Despite evidence supporting the relationship between online risk exposure and anxiety, the underlying mechanisms remain underexplored. This study aimed to construct and validate a chain-mediation structural equation model to investigate how online risk exposure influences anxiety among college students. Data was collected online in April 2023 from a sample of college students (*N* =  986, 64.6% female) using validated scales measuring online risk exposure, negative attribution, interpersonal security, and anxiety. The results showed that online risk exposure not only directly exacerbates anxiety but also indirectly affects it through three distinct pathways: negative attribution, interpersonal insecurity, and their chain relationship. These mediation effects accounted for 41.16%, 20.47%, and 4.65% of the total effects, respectively. These findings highlight the importance of targeted interventions aimed at reducing online risk exposure, improving negative attribution styles, and enhancing interpersonal security among college students. The study concludes by discussing its limitations and proposing directions for future research.

## 1. Introduction

Anxiety, which is a negative emotion experienced when individuals perceive potential negative consequences in risky situations [1], is notably characterized by an acute sense of apprehension, prevalent feelings of unease and concern, as well as maladaptive behaviors [2]. Indeed, moderate levels of anxiety can serve a crucial role by eliciting heightened alertness, augmenting concentration, and optimizing academic efficiency among college students, nevertheless, persistent and excessive anxiety can have a serious impact on daily life [3]. The prevalence of anxiety among college students has been on the rise, with rates reaching 13.7% over the past decade [4]. The etiology of anxiety is intricate and diverse, particularly in the era of “Internet+” and AI, where the effects of cyber information security and online risk activities on college students’ anxiety are becoming more prominent, with one study showing a significant correlation between online risk exposure and anxiety among college students [5]. To enhance the prevention and intervention of anxiety among college students, it is imperative for educators and students alike to focus on its genesis. Consequently, there is a critical need to delve into the inherent mechanisms mediating the relationship between online risk exposure and anxiety in college students.

Moreover, online risk exposure refers to the extent to which an individual directly experiences or encounters various negative risk situations while using the internet, which mainly includes four risk scenarios: information disclosure, cyberbullying, online sexual solicitation, and exposure to violent or explicit content [6,7], the prevalence rates within specific subgroups of college students are in order: 75.6% [8], 11.3% to 32.1% [9,10], 15.3% to 48.4% [11], and 28.6% to 90% [8,12]. Several studies have indicated that online risk activities significantly impact individuals’ emotional well-being [13–16], and may even precipitate self-harm and suicidal behaviors [10,17]. This underscores the high detection rate and severity of online risk exposure within the university student population, revealing that online risk exposure has emerged as a significant social issue and a novel threat to college students’ emotional health [5]. To our knowledge, however, no studies have examined in depth the mechanisms through which online risk exposure affects the anxiety of college students.

Expanding on prior studies, this research broadens the scope by incorporating negative attribution and interpersonal security as intermediary factors. This extension aims to address the questions of “how online risk exposure affects anxiety among college students” and “how to reduce anxiety caused by online risk exposure”. These insights are vital in aiding mental health professionals to develop more precise and impactful intervention tactics to mitigate the damage from online risk exposure.

## 2. Literature review and hypotheses establishment

### 2.1. The fundament theoretical framework

Lazarus, an American psychologist, introduced the cognitive-motivational-relational theory of emotion in 1991, positing a close interconnection and interaction between cognition, motivation, and emotion. Emotion is an individual’s response to meaning, i.e., how the individual makes sense of the stimulus event [18,19]. If a person views a stimulus event as having a positive or negative effect on him or her, a corresponding emotional or reactive inclination will emerge. Lazarus conducted a thorough examination of “cognitive appraisal” and “coping”, positing that whether an individual felt stressed when coping with emergencies hinged on these two principal psychological processes [20,21]. “Cognitive appraisal” refers to how people assess a situation’s effect on them, while “coping” involves managing the conflict between a person and the situation through actions or cognitive techniques, as well as this coping can bridge the gap between the risk events and the emotional outcomes. Emotional states change from the beginning to the end of the coping process. Concurrently, motivation plays an important role in the process of coping, and it can lead to either positive or negative emotional feedback effects. “Negative coping” can easily induce adverse emotional experiences and bring harm to individuals, while “positive coping” can help to resolve internal conflicts and benefit individuals, thus producing positive emotional experiences. Consequently, Lazarus’ cognitive-motivational-relational theory of emotion highlights the crucial role of cognitive appraisal and coping strategies in shaping individuals’ emotional reactions, pointing out that how an individual comprehends and responds to a stimulus event determines their positive or negative emotional experiences.

### 2.2. Online risk exposure and anxiety

Extensive researches had demonstrated the negative implications of online risks, such as cyberbullying and sexual solicitation, on individuals’ emotional well-being [14,16,17,22]. The latest research had established a positive correlation between online risk exposure and anxiety [5]. Lifestyle exposure theory (LET) posits that individuals’ likelihood of victimization increases with their extent of exposure to potential criminals and risky situations [23,24]. The variation in online lifestyles, behaviors, and interactions with unfamiliar social contacts can result in different levels of potential harm, explaining why some individuals may become victims of network crimes. For example, research has shown a correlation between online risk activities associated with lifestyle, leisure, and social networking, and the occurrence of online bullying among adolescents [25]. Besides, 45.8% of Chinese college students spend an average of three to six hours daily on their smartphones, mainly for recreational activities [26]. Considering that a significant proportion of Chinese college students spend several hours daily engaging in recreational activities on smartphones, it is evident that increased online activity can lead to greater exposure to potential risks. From the perspective of the LET, online risk exposure can be viewed as an environment that heightens the propensity for criminal activities, thereby increasing college students’ anxiety and vulnerability to victimization. In conclusion, we propose the first research hypothesis (**H1**): online risk exposure is positively correlated with anxiety levels among college students.

### 2.3. The role of negative attribution in online risk exposure and anxiety

Negative attribution refers to individuals’ tendency to interpret others’ attitudes, behaviors, or events negatively, even when these may not explicitly involve negativity [27]. Cognitive-emotional process model [28] suggest that attributing causality to specific entities or occurrences is crucial in shaping individuals’ emotional responses. Attribution, a cognitive procedure involving causal explanations for favorable and unfavorable outcomes [29], plays a pivotal role in this process. Individuals with positive attributions lean towards external, unstable, and specific explanations during adverse circumstances, while those with negative attributions tend to ascribe internal, stable, and universal interpretations [30].

According to stress-coping theory, cognitive appraisals serve as a bridge between stressful events and subsequent reactions [20,21]. Previous research has demonstrated that stress can lead individuals to adopt maladaptive attribution styles. For example, children exposed to adverse situations, such as victimization, frequently attribute these experiences to their own negative traits, with the severity of negative attributions positively correlated with the frequency of victimization. [31]. Attribution styles are closely linked to psychological well-being [32], with negative attribution leading to feelings of despair and a perceived lack of control over life events [33], which is a significant risk factor for anxiety. Additionally, there is a direct correlation between the negativity of attribution style and the intensity of anxiety [34].

Furthermore, negative attribution may function as a mediator in the association between online risk exposure and anxiety. According to the cognitive appraisal theory of emotion, individuals evaluate the perceived threat, challenge, or harm of an event, which elicits varied emotional responses and subsequently shapes their behavioral outcomes [18,19]. Cognitive appraisal acts as a mediating mechanism in the relationship between risk incidents and emotional outcomes, influencing an individual’s emotional state from the initial encounter to its resolution [20,21]. At the same time, empirical studies showed that risk exposure, especially sexual abuse, was directly, positively associated with negative attributions [35]. Consistent with this perspective, negative attribution, functioning as a cognitive appraisal process [29], may play a mediating role in the relationship between online risk exposure and anxiety.

Drawing from the above relevant studies, it can be inferred that negative attribution acts as a conduit between online risk exposure (the stimulus event) and anxiety (the ensuing response). Thus, we propose our second research hypothesis (**H2**): Negative attribution mediates the relationship between online risk exposure and anxiety.

### 2.4. The role of interpersonal security in online risk exposure and anxiety

Interpersonal security refers to the positive experience individuals derive from maintaining a favorable self-perception during interpersonal interactions [26]. It encompasses feelings of interpersonal harmony, optimism and cheerfulness, respect and friendship, as well as happiness and warmth in relationships [25]. More importantly, interpersonal security is not only a fundamental aspect of overall security [24, 25], but also plays a crucial role in the formation of interpersonal relationships [39]. Individuals who possess a sense of interpersonal security feel at ease, confident, and fulfilled in their interactions with others, enabling them to cultivate lasting relationships without succumbing to excessive unease or anxiety during environmental shifts; Conversely, individuals lacking interpersonal security are more prone to questioning the benevolence of others [40], which can lead to lower self-esteem, emotional distress [41,42], and a greater inclination towards adverse behaviors [43]. Notably, low interpersonal security has been linked to suicidal ideation [44]. Taken together, interpersonal security is a positive and important experience derived from self-perception rooted in a sense of acceptance and belonging during social interaction, while its absence can lead to maladaptive outcomes, such as emotional distress (e.g., anxiety), and problem behaviors (e.g., social avoidance) [45].

Furthermore, interpersonal security may also play an important role between online risk exposure and anxiety. On the one hand, online risk exposure, as a negative and complex social interaction situation, can contribute to interpersonal suspicion and insecurity. There is some evidence that individuals frequently grappled with profound insecurity when confronted by risks or losses [28]. Specifically, breaches of private information seriously undermine interpersonal security and victims develop distrust of others as a result of the invasion of their privacy, and this distrust leads to anxiety and unease in individuals, making it difficult for them to establish stable interpersonal relationships [13,46]. Cyberbullying has a profoundly destructive effect on the interpersonal security, not only leading to isolation, social segregation and damage to the victim’s self-esteem, but also potentially triggering anxiety, depression and even suicidal tendencies [45]. Its persistence and anonymity make it difficult for victims to escape, thus deepening their fear and insecurity of interpersonal interactions [13]. Online sexual solicitation profoundly affects interpersonal security by violating physical and psychological boundaries, causing excessive suspicion and distrust, and leading to social avoidance due to feelings of shame and fear [47,48]. On the other hand, interpersonal security is an important variable influencing anxiety [49]. Empirical studies have also shown a negative correlation between interpersonal security and anxiety among college students [40,41]. Thus, it was further hypothesized that interpersonal security would mediate the relationship between online risk exposure and anxiety (**H3**).

### 2.5. The role of negative attribution and interpersonal security in online risk exposure and anxiety

Individuals often resort to negative causal attributions when confronted with unforeseen adverse events [42]. Emotional abuse has been linked to a pervasive negative attribution style and cognitive appraisal, including inferring negativity from neutral events [43]. It can be inferred that online risk exposure, as a negative experience, causes a strong psychological shock and even emotional abuse to the individual, which in turn triggers negative cognitive appraisal processes, such as negative attribution.

More importantly, relevant studies have suggested that negative attribution corresponds to lower levels of interpersonal security among college students [37,44]. Attribution style negatively influences interpersonal security, indicating that a more negative attribution style is associated with lower levels of interpersonal security among college students [37]. Additionally, individuals with insecurity often tend to attribute others’ behavior to more negative factors [35,44]. Specifically speaking, negative attributions disrupt interpersonal harmony by undermining interpersonal trust, and further increase interpersonal anxiety and uneasy [39]. It can also lead to more negative evaluations of other people’s negative behaviors, induce pessimistic attitudes towards risks and challenges [35,44]. In addition, self-esteem can be damaged, particularly when individuals attribute others’ adverse behavior to their own shortcomings [34]. Ultimately, such attributions can increase suspicion of others’ motivations, leading to isolation and indifference, and a lack of warm emotional support, which can reduce well-being [50]. These studies suggest a closely relationship between negative attribution, interpersonal insecurity and anxiety.

Even more importantly, according to the cognitive-motivational-relational theory of emotion, when individuals appraisal online risk exposures to be hurtful, threatening, or challenging to them, more likely to induce individuals’ negative attribution, failure to respond effectively to risks may affect their interpersonal security, which can further lead to negative emotions such as anxiety and depression or maladaptive behavioral responses such as avoidance and violence [19,51,52]. Given this evidence, we propose the fourth research hypothesis (**H4**): Negative attribution and interpersonal security play a chained mediating role between online risk exposure and anxiety.

**Fig 1:**
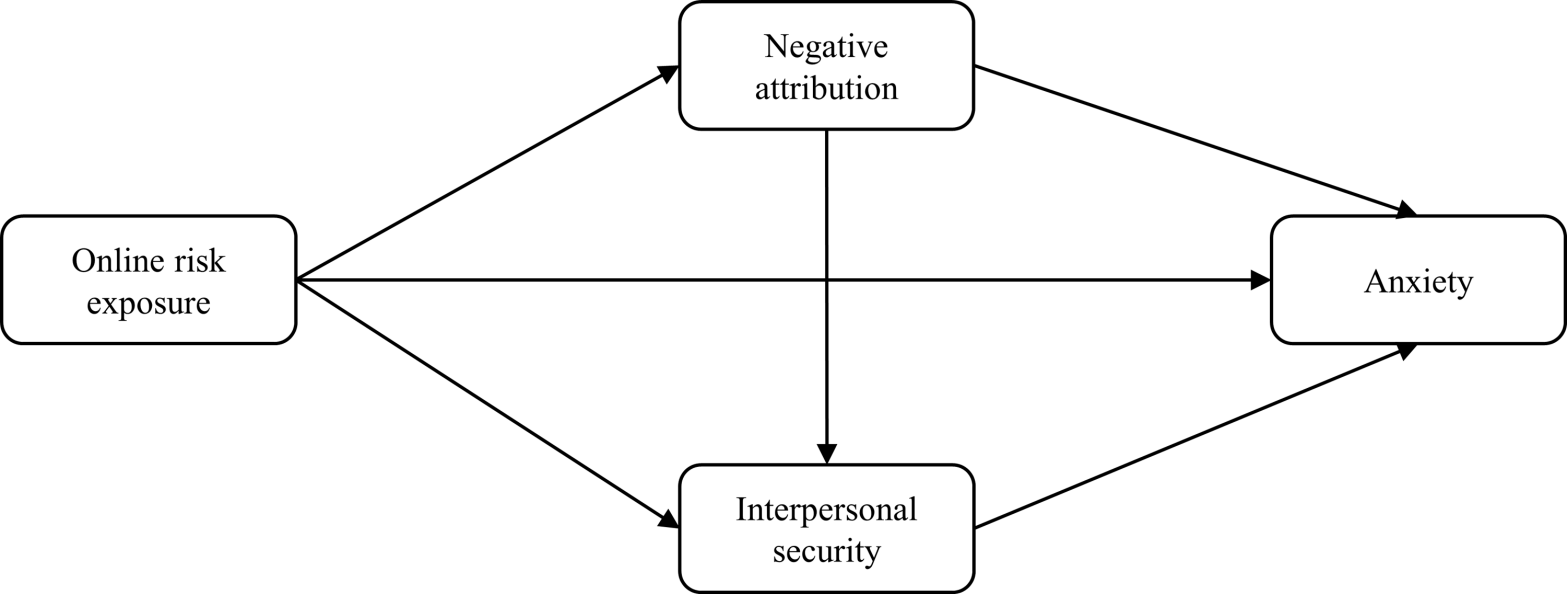
Research hypothetical model.

To sum up, building on the cognitive-motivational-relational theory of emotion and supported by relevant empirical studies, a chain-mediation model was developed to investigate the mechanisms underlying the relationship between online risk exposure and anxiety. Specifically, the single mediating role of negative attributions, interpersonal safety, and their chain mediating role are examined. In doing so, this study extends our understanding of the relationship between online risk exposure and anxiety by shedding light on the internal mechanism. Furthermore, it would provide theoretical guidance and empirical support for promoting emotional health among college students. Finally, the hypothetical model is shown in Fig.1.

## 3. Methods

### 3.1. Participants and procedure

Approval of this research was obtained from the ethics committee of the key laboratory of modern teaching technology ministry education in Shaanxi Normal University with reference number: L20230410-01. In this study, a stratified sampling method was employed to survey undergraduate students from two comprehensive universities located in midwestern China. One is located in Xi’an, Shaanxi Province and the other in Yichang, Hubei Province. All participants provided electronic version of the informed consent prior to taking part in the study. Data was collected through an online questionnaire distributed via the Star questionnaire platform (https://www.wjx.cn) in April 2023. Participants accessed the questionnaire by scanning a provided QR code with their mobile phones and were required to submit their responses within a 10-minute time frame. After excluding 64 invalid questionnaires due to careless responses, such as choosing answers based on meaningless patterns [53] or straight-lining & nondifferentiation responses [54,55], a total of 986 usable questionnaires were obtained (*M*_*age*_ =  19.73, *SD* =  1.01), resulting in an effectiveness rate of 93.90%. The sample consisted of both male (*n* =  349, 35.40%, *M*_*age*_ =  19.44, *SD* =  1.25) and female (*n* =  637, 64.60%, *M*_*age*_ =  19.88, *SD* =  0.81) participants, including freshmen (*n* =  483, 48.99%), sophomores (*n* =  271, 27.48%), and juniors (*n* =  235, 23.83%).

### 3.2. Measures

#### 3.2.1. Online risk exposure scale.

The online risk exposure scale, initially developed by Wisniewski et al [6] and subsequently adapted for Chinese contexts by Zhang et al. [5], consists of 16 items distributed across four sub-scales: information breaches (3 items, such as, “I shared my personal information or a photo of myself that I later regretted sharing.”), cyberbullying (4 items), online sexual solicitations (4 items), and exposure to explicit content (5 items). Participants rate each item on a 5-point Likert scale ranging from 1 (none) to 5 (a lot), indicating the extent of their exposure to online risks. Higher arithmetic average scores indicate greater exposure to online risk. The reliability analysis indicated satisfactory internal consistency for the scale in the current sample, with a *Cronbach’s Alpha* of 0.933 and a Kaiser-Meyer-Olkin (*KMO*) value of 0.938. The *Cronbach’s Alpha* for each sub-scale were 0.800, 0.895, 0.925, and 0.901, respectively.

#### 3.2.2. Anxiety scale.

The anxiety scale from the depression, anxiety, and stress scale-21 (DASS-21), originally developed by Lovibond et al. [56] and later adapted for the Chinese context by [57], was used in this study. This sub-scale comprises 7 items measuring anxiety in a single dimension and utilizes a Likert four-point scale. The response options range from 0 to 3, indicating “non-conformance” to “constant conformance”, e.g., “I was scared for no reason”. The scores are calculated by obtaining the arithmetic mean of all items, with higher scores indicating higher levels of anxiety among college students. Reliability analysis demonstrated satisfactory internal consistency for the scale in the current sample, with a *Cronbach’s Alpha* of 0.870 and a *KMO* value of 0.905.

#### 3.2.3. Negative attribution scale.

The negative attribution sub-scale used in this study was adapted from the positive-negative ruminative thinking scale developed by Yang et al. [58]. This sub-scale consists of 5 items, e.g., “Think ‘Bad just turns to worse’”, and utilizes a Likert four-point scale, with response options ranging from 1 to 4, representing “never” to “always”. A higher arithmetic mean score indicates a greater inclination towards negative attribution. The scale demonstrated satisfactory internal consistency in the current sample, with a *Cronbach’s Alpha* of 0.871 and a *KMO* value of 0.855.

#### 3.2.4. Interpersonal security scale.

The interpersonal security scale used in this study was adapted from Maslow’s security-insecurity questionnaire [59] and subsequently modified for Chinese contexts by Cao et al. [36]. It comprises 18 items divided into four dimensions: interpersonal harmony (5 items, e.g., “I get along well with people of the opposite sex.”), optimism and cheerfulness (3 items), respect and friendship (4 items), as well as happiness and warmth (6 items). Each item offers three response options: “yes”, “no”, and “not sure”, which are scored as “0”, “1” and “0” according to the provided score sheet. A higher arithmetic mean score indicates a greater sense of interpersonal security. Reliability analysis demonstrated satisfactory internal consistency for the scale in the current sample, with a *Cronbach’s Alpha* of 0.934 and a *KMO* value of 0.937. The *Cronbach’s Alpha* for each dimension were 0.798, 0.862, 0.813, and 0.909, respectively.

### 3.3. Data analyses

Firstly, invalid questionnaires were filtered out according to the methods of inaccurate or regular responses, as well as using the boxplot test. Secondly, *SPSS version 22.0* was utilized to conduct the common method deviation test, reliability analysis, descriptive statistics, and correlation analysis. Thirdly, the structural equation modeling (SEM) was constructed and tested using *AMOS version 24.0* to examine the influence effects of negative attribution and interpersonal security on the relationship between online risk exposure and anxiety. The ideal ranges for the model goodness-of-fit indices are as follows: the *RMSEA* should be less than 0.08, the *χ2/df* should not exceed 3, the *TLI* and *CFI* should be higher than 0.9, as well as the *SRMR* below 0.05 [60]. Additionally, it is important to note that the effect ratio represents the percentage of the direct or indirect effect value in relation to the total effect value of the model. Moreover, modifications to the model were made by incorporating the covariance of error terms within the same factor based on the suggestions provided by the “modification indices”.

## 4. Results

### 4.1. Test for common method bias

As the data in this study were collected through self-reports from college students, the possibility of common method bias was considered. To address this concern, an unrotated principal component analysis was conducted on all variables using Harman’s single-factor test [61]. The results indicated that all seven factors had characteristic root values greater than 1, and the first principal factor explained 30.699% of the variance, which is below the threshold of 40%. These findings suggest that common method bias is not a significant concern in this study.

### 4.2. Descriptive statistics and correlation analysis

The Pearson correlations presented in Table 1 revealed a significant correlation between each variable and its internal factors. These correlations provide initial evidence supporting the mediation role of negative attribution and interpersonal security in the relationship between online risk exposure and anxiety.

**Table 1:** Descriptive statistics and Pearson’s correlation coefficient (N =  986).

	*M ± SD*	1	2	3	4	5	6	7	8	9	10	11
1 Anxiety	1.61 ± 0.541	1										
2 Interpersonal security	0.69 ± 0.314	-0.495***	1									
3 Interpersonal harmony	0.59 ± 0.352	-0.418***	0.797***	1								
4 Optimism and cheerfulness	0.69 ± 0.410	-0.409***	0.876***	0.577***	1							
5 Respect and friendship	0.72 ± 0.352	-0.429***	0.895***	0.654***	0.705***	1						
6 Happiness and warmth	0.75 ± 0.357	-0.436***	0.843***	0.510***	0.667***	0.706***	1					
7 Negative attribution	2.18 ± 0.646	0.584***	-0.282***	-0.236***	-0.254***	-0.230***	-0.241***	1				
8 Online risk exposure	1.55 ± 0.581	0.483***	-0.325***	-0.239***	-0.280***	-0.300***	-0.290***	0.326***	1			
9 Information breaches	1.85 ± 0.824	0.395***	-0.241***	-0.185***	-0.221***	-0.214***	-0.199***	0.286***	0.802***	1		
10 Cyberbullying	1.43 ± 0.669	0.412***	-0.312***	-0.232***	-0.255***	-0.299***	-0.283***	0.249***	0.891***	0.661***	1	
11 Online sexual solicitations	1.25 ± 0.582	0.401***	-0.285***	-0.207***	-0.231***	-0.257***	-0.278***	0.215***	0.845***	0.511***	0.767***	1
12 Exposure to explicit content	1.68 ± 0.744	0.388***	-0.245***	-0.172***	-0.219***	-0.230***	-0.213***	0.309***	0.774***	0.406***	0.554***	0.603***

* *p* < 0.05, ^**^p < 0.01, ^***^p < 0.001, same as below.

### 4.3. Mediation analysis

To preliminarily verify hypothesis H1, stepwise regression analysis was conducted with online risk exposure as the independent variable and anxiety as the dependent variable. The regression analysis (see Table 2) indicated that all components of online risk exposure can significantly affect anxiety levels, implying that an increase in online risk exposure can result in elevated levels of anxiety in college students (L4: *F* = 75.303, *R*^*2*^ = 0.235, *β*_*1*_ = 0.078, *p*_*1*_ < 0. 001; *β*_*2*_ = 0.186, *p*_*2*_ < 0. 001; *β*_*3*_ = 0.204, *p*_*3*_ < 0. 001; *β*_*4*_ = 0.125, *p*_*4*_ < 0. 001).

**Table 2:** Stepwise regression analysis of online risk exposure on anxiety (*N* =  986).

Dependent variable	Independent variables	L1	L2	L3	L4
Anxiety	Cyberbullying	0.412***	0.284***	0.156***	0.078***
	Exposure to explicit content		0.230***	0.219***	0.186***
	Information breaches			0.203***	0.204***
	Online sexual solicitations				0.125***
	*R* ^*2*^	0.169	0.206	0.229	0.235
	Adjusted *R*^*2*^	0.169	0.205	0.227	0.232
	*F*	200.735***	127.620***	97.355***	75.303***

L1-L4, Stepwise regression model.

To further validate research hypotheses H1, H2, H3, and H4, a stepwise methodology [62] was employed to construct models. Sequentially, negative attribution and interpersonal security were added to establish model M1 without mediation, followed by model M2 and M3 with single mediation, model M4 with chain mediation, and model M5 with chain-multiple mediation (Table 3). Model M2 exhibited a *SRMR* value exceeding 0.05, prompting modifications to generate model M2a by incorporating a covariant correlation of error variables within the same factor using the information from “modification indices”. Significance of parameters was determined using Bootstrap self-sampling (2000 samples) with a 95% confidence interval (CI) [Lower, Upper], excluding zero [63]. The data presented in Table 3 indicated that each model demonstrated good fit based on various fit indicators.

**Table 3:** Significance test and effect value of each model fitness indices and mediation effect (N =  986).

Model	*χ2/df*	*CFI*	*TLI*	*RMSEA*	*SRMR*	Pathways	*B*	95%*CI* [Lower, Upper]	*B*%
M1	5.921	0.960	0.947	0.071	0.035	① → ②	0.446	[0.369, 0.522]	100
M2	5.293	0.946	0.936	0.066	0.051	① → ②	0.243	[0.185, 0.307]	54.98
						① → ③ → ②	0.199	[0.152, 0.254]	45.02
M2a	4.788	0.954	0.944	0.062	0.048	① → ②	0.236	[0.178, 0.301]	54.13
						① → ③ → ②	0.199	[0.153, 0.254]	45.64
M3	4.921	0.955	0.945	0.063	0.045	① → ②	0.279	[0.205, 0.357]	64.73
						① → ④ → ②	0.151	[0.112, 0.201]	35.03
M4	6.536	0.914	0.900	0.075	0.042	① → ②	0.297	[0.221, 0.375]	85.10
						① → ③ → ④ → ②	0.053	[0.034, 0.080]	15.19
M5	3.949	0.954	0.947	0.055	0.046	① → ②	0.146	[0.087, 0.206]	33.95
						① → ③ → ②	0.177	[0.135, 0.228]	41.16
						① → ④ → ②	0.088	[0.060, 0.123]	20.47
						① → ③ → ④ → ②	0.020	[0.012, 0.031]	4.65

①= online risk exposure, ②= anxiety, ③= negative attribution, ④= interpersonal security.

**Fig 2:**
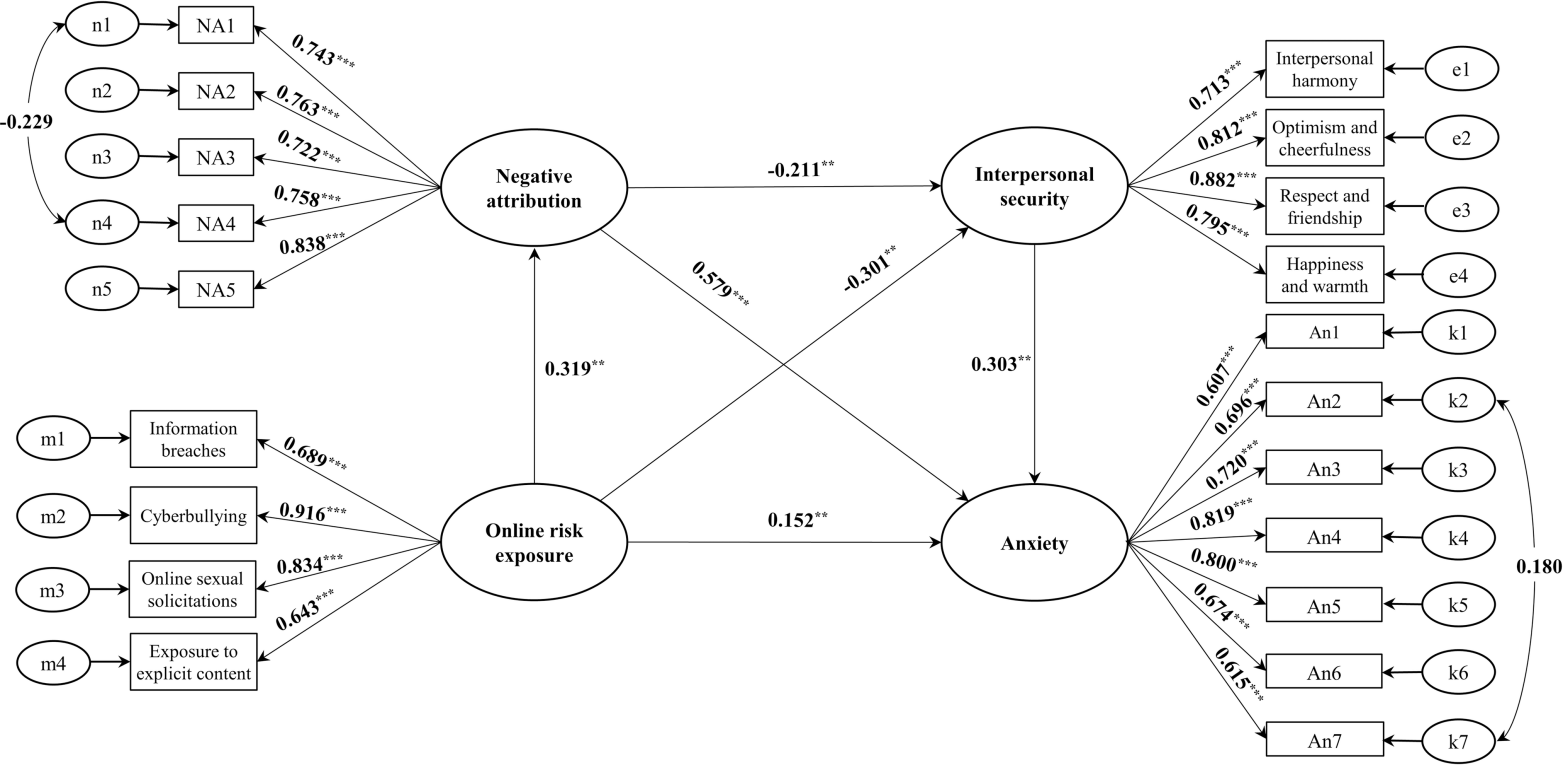
The multi-mediating model M5 of negative attribution and interpersonal security in online risk exposure and anxiety (N=  986).

The model M5 (Fig.2) suggested a well-fitted model (Table 3). We found that online risk exposure is positively correlated with negative attribution and anxiety (*β* =  0.319, *p* <  0.01; *β* =  0.152, *p* <  0.01), as well as negatively correlated with interpersonal security (*β* =  -0.211, *p* <  0.01). In addition, negative attribution is correlated positively with anxiety (*β* =  0.579, *p* <  0.001) and negatively with interpersonal security (*β* =  -0.211, *p* <  0.01), which is correlated negatively with anxiety (*β* =  -0.303, *p* <  0.01). A bootstrap test was used to found that the direct effect of online risk exposure and anxiety was 0.146, accounting for 33.95% of the total effect, with *CI* =  [0.087, 0.206]. Negative attributions were identified as a significant mediator between online risk exposure and anxiety (*B* =  0.177, *CI* =  [0.135, 0.228]), with the mediation effect accounting for 41.16% of the total effect. Furthermore, the mediation effect of interpersonal security in the relationship between online risk exposure and anxiety was significant (*B* =  0.088, *CI* =  [0.060, 0.123]), with the mediation effect accounting for 20.47% of the total effect. The chain mediation effect of negative attribution and interpersonal security in the relationship between online risk exposure and anxiety was 0.020, accounting for 4.65% of the total effect, with *CI* =  [0.012, 0.031]. These findings suggest that negative attributions and interpersonal security play a chained mediating role in the impact of online risk exposure on anxiety.

## 5. Discussion

### 5.1. Online risk exposure and anxiety

Our research confirms that online risk exposure has a positive influencing effect on anxiety among college students. This finding substantiates hypothesis H1, which aligns with earlier studies [5,14]. This study finds that cyberbullying, online sexual solicitation, private information breaches and exposure to explicit content negatively affect college students’ anxiety, further indicating that online risk exposure can trigger different degrees of anxiety. Specifically, private information breaches can threaten individual security and trigger anxiety and uneasy [64]. Cyberbullying can cause recurring bullying-related words or images in the individual’s mind, and this repeated rumination on bullying incidents weakens the individual’s emotional regulation ability, thereby heightening negative emotions such as anxiety and depression [14]. Online sexual solicitation can amplify an individual’s anxiety, through the continuous increase of negative information, the pessimistic expectations of future, and the intensification of self-doubt [48]. And exposure to explicit content can trigger negative emotion, leading to uneasy and mistrust, which further induce anxiety [51]. Therefore, cyberbullying, online sexual solicitation, breaches of private information and exposure to explicit content are all factors that contribute to the onset and worsening of anxiety. And prevention of online risk exposure among college students can focus on protecting privacy, promoting the dangers of online bullying, providing online sexuality education, and cleaning up cyberspace.

### 5.2. The mediating role of negative attribution

The results support hypothesis H2 which holds that negative attribution plays a mediating effect between online risk exposure and anxiety among college students. This finding aligns with previous research outcomes suggesting that risk exposure positively impacts individual anxiety through negative attribution [31,33,38]. The results of this study can be supported by stress coping theory and attribution theory. Stress coping theory emphasizes individuals’ cognitive appraisal and coping strategies when facing stressors. Online risk exposure (such as privacy breaches and cyberbullying) is seen as a stressor. If individuals assess that they cannot cope with these risks, anxiety may arise [20,31]. In addition, attribution theory focuses on how individuals explain the causes of events and their impact on emotions and behaviors. Individuals with a negative attribution style tend to attribute adverse events to internal, stable, and uncontrollable factors [33]. Therefore, if individuals attribute online risks to their own traits (e.g., “I’m too stupid”) rather than external factors (e.g., “The online environment is complex”), they are more likely to experience anxiety [34].

In addition, according to Seligman’s research, one person is positive and optimistic because he often attributes negative events and emotions to external, temporary factors while attributing positive events and emotions to internal, stable factors [65]. Therefore, the significance of this study lies not only in illuminating the mechanisms underlying anxiety in college students but also for advocating the cultivation of a positive attribution style among students.

### 5.3. The mediating role of interpersonal security

This study found that interpersonal security functions as a mediator between online risk exposure and anxiety. As the level of online risk exposure increases, interpersonal security diminishes, resulting in heightened anxiety levels, thus confirming hypothesis H3. These findings align with previous research examining the link between reduced interpersonal security and emotional distress [40,41,66]. This mediating effect can be explained by attachment theory. Attachment style significantly influences how individuals perceive and respond to others’ behavior in situations of stress and uncertainty (e.g., online risk exposure). Interpersonal security plays a key role in this process. Individuals with secure attachment styles typically show higher levels of trust and emotional stability, whereas individuals with insecure attachment styles are prone to anxiety and mistrust, which can lead to increased skepticism of others’ motives and social avoidance.

There is also empirical research that can explain the findings of this study. For example, experiences such as online sexual solicitation or cyberbullying can lead to feelings of insecurity due to the potential loss of benefits [67], which can affect interpersonal trust and fuel anxiety. The finding of this study is of great value. On the one hand, this study highlights the importance for college students to recognize their importance and value in their interpersonal relationships. On the other hand, institutional trust indirectly strengthens interpersonal trust among strangers by fostering a sense of security [68]. These insights highlight the need for comprehensive cybersecurity policies to monitor and reduce online risks, as well as strengthen interpersonal trust in institution.

### 5.4. The chain mediating role of negative attribution and interpersonal security

Following the confirmation of H2 and H3, the research findings further confirm H4: negative attribution and interpersonal security play a chain-multiple mediating effect between online risk exposure and anxiety. The mediation process suggests that online risk exposure influences individuals’ negative attribution of risk perception, which subsequently reduces interpersonal security, ultimately leading to elevated levels of anxiety among college students. These findings align with previous studies [5,38,43]. Attachment theory also provides insights into these relationships, as insecure attachment is strongly linked to interpersonal insecurity and various maladaptive strategies, including negative attribution [69]. Moreover, negative coping strategies are associated with a sense of security, impacting interpersonal security and thus influencing social anxiety [58]. Additionally, the use of social networking sites has shown a negative correlation with markers of secure attachment and a positive correlation with attachment anxiety [70]. These studies partially support the findings of our research.

Based on the discussion of H2 and H3, this study further adds that while encountering online risks exposure, some emotional problems involve both the “how to influence anxiety” and the “how to reduce anxiety”, and the process of solving them requires a chain-effect of negative attribution and interpersonal security. The mediation effects of negative attribution, interpersonal insecurity, and their chain relationship accounted for 41.16%, 20.47%, and 4.65% of the total effects, respectively. These ratios suggest that exposure to online risks makes negative attribution the primary cause of anxiety and interpersonal insecurity, with interpersonal insecurity serving as an important mediator. While the chain-effect of negative attribution and interpersonal insecurity constitutes a small portion of the overall effect, its statistical significance highlights its practical implications. This chain-effect represents the degree to which negative attributions influence interpersonal security. In particular, individuals prone to negative attribution are more likely to experience heightened interpersonal insecurity, contributing to anxiety and emotional distress [37]. In this instance, employing positive attribution and bolstering interpersonal security are required to alleviate these challenges for college students, thereby promoting the continuous development of their emotional health.

## 6. Conclusions, limitations and implications

This research delved into how online risk exposure affects anxiety based on the cognitive-motivational-relational theory of emotion and literature research, constructed a multi-intermediary structural equation model and performed validation studies on it to further investigate the relationship between “how to influence anxiety” and “how to reduce anxiety”. The findings of this study not only highlight the important mediating role of negative attribution and interpersonal security in the relationship between online risk exposure and anxiety among college students, but also emphasize the importance of targeted interventions. Interventions should focus on reducing online risk exposure, improving negative attributive styles, and potentially strengthening interpersonal security in college students.

This study, like any other, is not without limitations. First, the cross-sectional design limits the ability to establish temporal relationships, potentially obscuring the dynamic effects of online risk exposure on anxiety levels. should employ longitudinal approaches to better capture the temporal evolution and causal relationships among these variables. Second, this study is subject to certain limitations in terms of the selection of the sampled population. Due to senior students being preoccupied with job hunting and graduation preparations during the survey period, they were not included in the sample. This limits the external validity of the research conclusions. Future studies could consider including senior students and graduate students to enhance the generalizability of the conclusions.

In addition, this research systematically explores the multi-mediating role of negative attribution and interpersonal security in the relationship between online risk exposure and anxiety. Unraveling these relationships has significant implications. On one hand, this study can provide a new perspective on the psychological mechanisms behind anxiety, further expanding the theoretical framework for research on online behavior and mental health. On the other hand, this study can provide important practice guidance for the construction and implementation of online mental health intervention program, particularly in terms of intervention pathways to reduce anxiety by promoting positive attributional styles and enhancing interpersonal security online.

## Supporting information

S1 Data Minimal dataset. (XLSX)
