# Correction: Online risk exposure and anxiety among college students in China: The chain mediating role of negative attribution and interpersonal security

**DOI:** 10.1371/journal.pone.0322421

**Published:** 2025-04-07

**Authors:** The PLOS One Editors

## Notice of republication

This article [1] was republished on March 24, 2025, to address an issue identified post-publication and to provide an updated version of S1 Data. Please download this article again to view the correct version.
